# Preservation Rhinoplasty Pros and Cons: An Expert Video Roundtable

**DOI:** 10.1093/asjof/ojaf092

**Published:** 2025-07-09

**Authors:** Jamil Ahmad, Jason Roostaeian, Aaron M Kosins, T Jon Kurkjian

The authors are pleased to present an expert roundtable discussion on preservation rhinoplasty (Video). The discussants include a panel of leading experts in aesthetic surgery, and the video roundtable was recorded live at The Aesthetic MEET 2025 in Austin, TX. The panelists discussed the history of preservation rhinoplasty and how it represents a new approach to rhinoplasty compared with open structure rhinoplasty, a technique that the panelists all agreed was standard for them in their training. They each discussed their process of incorporating preservation rhinoplasty into their practice and the ways a surgeon can “ease in” to the preservation approach. They discussed the various ways to implement techniques, such as ultrasound rhinoplasty, and the benefits of using a “hybrid” approach of older and new rhinoplasty techniques that can be tailored to each patient's nose. The discussants concluded by covering the changing approaches to the lower part of the nose and how they have customized their techniques based on years of experience.

